# Cohort profile: the Turin prostate cancer prognostication (TPCP) cohort

**DOI:** 10.3389/fonc.2023.1242639

**Published:** 2023-10-06

**Authors:** Nicolas Destefanis, Valentina Fiano, Lorenzo Milani, Paolo Vasapolli, Michelangelo Fiorentino, Francesca Giunchi, Luca Lianas, Mauro Del Rio, Francesca Frexia, Luca Pireddu, Luca Molinaro, Paola Cassoni, Mauro Giulio Papotti, Paolo Gontero, Giorgio Calleris, Marco Oderda, Umberto Ricardi, Giuseppe Carlo Iorio, Piero Fariselli, Elena Isaevska, Olof Akre, Renata Zelic, Andreas Pettersson, Daniela Zugna, Lorenzo Richiardi

**Affiliations:** ^1^ Cancer Epidemiology Unit, Department of Medical Sciences, University of Turin, Turin, Italy; ^2^ DIMEC Department of Medicine and Surgery, Alma Mater Studiorum, University of Bologna, Bologna, Italy; ^3^ Department of Pathology, IRCCS Azienda Ospedaliero-Universitaria di Bologna, Bologna, Italy; ^4^ Visual and Data-intensive Computing, CRS4 (Center for Advanced Studies, Research and Development in Sardinia), Pula, Italy; ^5^ Pathology Unit, Department of Medical Sciences, University of Turin, Turin, Italy; ^6^ Pathology Unit, Department of Oncology, University of Turin, Turin, Italy; ^7^ Urology Unit, Department of Surgical Sciences, University of Turin, Molinette Hospital, Turin, Italy; ^8^ Department of Oncology, University of Turin, Turin, Italy; ^9^ Computational Biomedicine Unit, Department of Medical Sciences, University of Turin, Turin, Italy; ^10^ Department of Molecular Medicine and Surgery, Section of Urology, Karolinska Institutet, Stockholm, Sweden; ^11^ Department of Molecular Medicine and Surgery, Karolinska Institutet and Department of Pelvic Cancer, Karolinska University Hospital, Stockholm, Sweden; ^12^ Clinical Epidemiology Division, Department of Medicine Solna, Karolinska Institutet, Stockholm, Sweden

**Keywords:** prostate cancer, prognosis, prognostic modelling, digital pathology, DNA methylation

## Abstract

**Introduction:**

Prostate cancer (PCa) is the most frequent tumor among men in Europe and has both indolent and aggressive forms. There are several treatment options, the choice of which depends on multiple factors. To further improve current prognostication models, we established the Turin Prostate Cancer Prognostication (TPCP) cohort, an Italian retrospective biopsy cohort of patients with PCa and long-term follow-up. This work presents this new cohort with its main characteristics and the distributions of some of its core variables, along with its potential contributions to PCa research.

**Methods:**

The TPCP cohort includes consecutive non-metastatic patients with first positive biopsy for PCa performed between 2008 and 2013 at the main hospital in Turin, Italy. The follow-up ended on December 31^st^ 2021. The primary outcome is the occurrence of metastasis; death from PCa and overall mortality are the secondary outcomes. In addition to numerous clinical variables, the study’s prognostic variables include histopathologic information assigned by a centralized uropathology review using a digital pathology software system specialized for the study of PCa, tumor DNA methylation in candidate genes, and features extracted from digitized slide images via Deep Neural Networks.

**Results:**

The cohort includes 891 patients followed-up for a median time of 10 years. During this period, 97 patients had progression to metastatic disease and 301 died; of these, 56 died from PCa. In total, 65.3% of the cohort has a Gleason score less than or equal to 3 + 4, and 44.5% has a clinical stage cT1. Consistent with previous studies, age and clinical stage at diagnosis are important prognostic factors: the crude cumulative incidence of metastatic disease during the 14-years of follow-up increases from 9.1% among patients younger than 64 to 16.2% for patients in the age group of 75-84, and from 6.1% for cT1 stage to 27.9% in cT3 stage.

**Discussion:**

This study stands to be an important resource for updating existing prognostic models for PCa on an Italian cohort. In addition, the integrated collection of multi-modal data will allow development and/or validation of new models including new histopathological, digital, and molecular markers, with the goal of better directing clinical decisions to manage patients with PCa.

## 1 Introduction

Prostate cancer (PCa) is a major public concern: in 2020 it was the most common tumor among men in Europe, with over 470,000 diagnosed cases (1). It is a heterogeneous disease including both indolent and aggressive tumors, with different treatment options ranging from active surveillance, focal or radical treatment, systemic therapies, to palliation (2). Radical therapy may come at the cost of side effects, including incontinence and impotence (3). Therefore, the best treatment option should balance the risk of disease progression and death, and the severity of possible treatment side-effects, taking into account the life expectancy of patients and their quality of life. For these reasons, pre-treatment prognostication is an essential component of the clinical management of PCa that should safely direct the more radical curative measures towards high-risk patients and avoid over-treating those with indolent tumors.

There are only a few validated predictive models (in the form of risk-stratification tools, nomograms, and scores) to guide treatment decisions at the time of the initial diagnosis. Moreover, even when they are available, these models are rarely tuned for the routine use in the clinical settings in which they are to be employed (4). In 2019, a systematic review urged for the development of new models built on long-term survival outcomes while simultaneously considering competing risks (5).

In the current clinical practice, the most widely used tool for pre-treatment risk assessment is the D’Amico classification system (and its derivatives) (6), which classifies patients into low-, intermediate- and high-risk groups based on combinations of three core variables: Gleason score, clinical stage, and *prostate-specific antigen* (PSA) levels. Besides the D’Amico classification system, numerous pre-treatment risk stratification tools are available for PCa, including the *Cancer of the Prostate Risk Assessment* (CAPRA) score (7) and the *Memorial Sloan Kettering Cancer Centre* (MSKCC) nomogram (8). In a head-to-head comparison with other available nomograms and scores [including the National Institute for Health and Care Excellence (9), the American Urological Association (10), the European Association of Urology (2), the National Comprehensive Cancer Network (11), and the Cambridge Prognostic Groups (12)] performed on the Swedish population, the CAPRA and MSKCC models were superior to the other evaluated options in terms of discrimination for PCa-specific mortality (13), with a C-index at 10-years of follow-up of 0.80 (95% CI: [0.79, 0.81]) and 0.81 (95% CI: [0.80, 0.81]), respectively. These models build on the D’Amico model by extending its core variables with additional prognostic markers (including the patient’s age and either the number or the proportion of positive and negative cores) and they attempt to use — wherever possible — the whole range of values rather than stratifying the patients in strict risk groups.

Nevertheless, there exists ample opportunity to further improve prognostication models, for instance through the adoption of molecular markers (14–17) and computer-aided pathology (18). The development, validation, and calibration of better prognostic models for PCa is an active area of research that could improve both survival and quality of life (19).

We established the *Turin Prostate Cancer Prognostication* (TPCP) cohort, a historical biopsy cohort of approximately 900 unselected consecutive PCa patients diagnosed between 2008 and 2013 at the “*A.O.U. Città della Salute e della Scienza di Torino*” (hereafter referred to as “University Hospital”), the main hospital of the city of Turin, Italy, with the aim to recalibrate and revise existing prognostic models to inform the clinical decision-making for PCa (20). Furthermore, we aim to exploit the new data to improve the existing models through advanced statistical modelling and the inclusion of new prognostic variables, such as novel histopathological features extracted from digitized slides, and tumor tissue DNA methylation markers. Tumor DNA will also be biobanked for future analyses.

Here we describe the main characteristics of the TPCP cohort in terms of study design, patient outcomes, planned data and molecular analyses, and availability of clinical and non-clinical information at diagnosis and during follow-up.

## 2 Methods and study design

**Figure 1:**
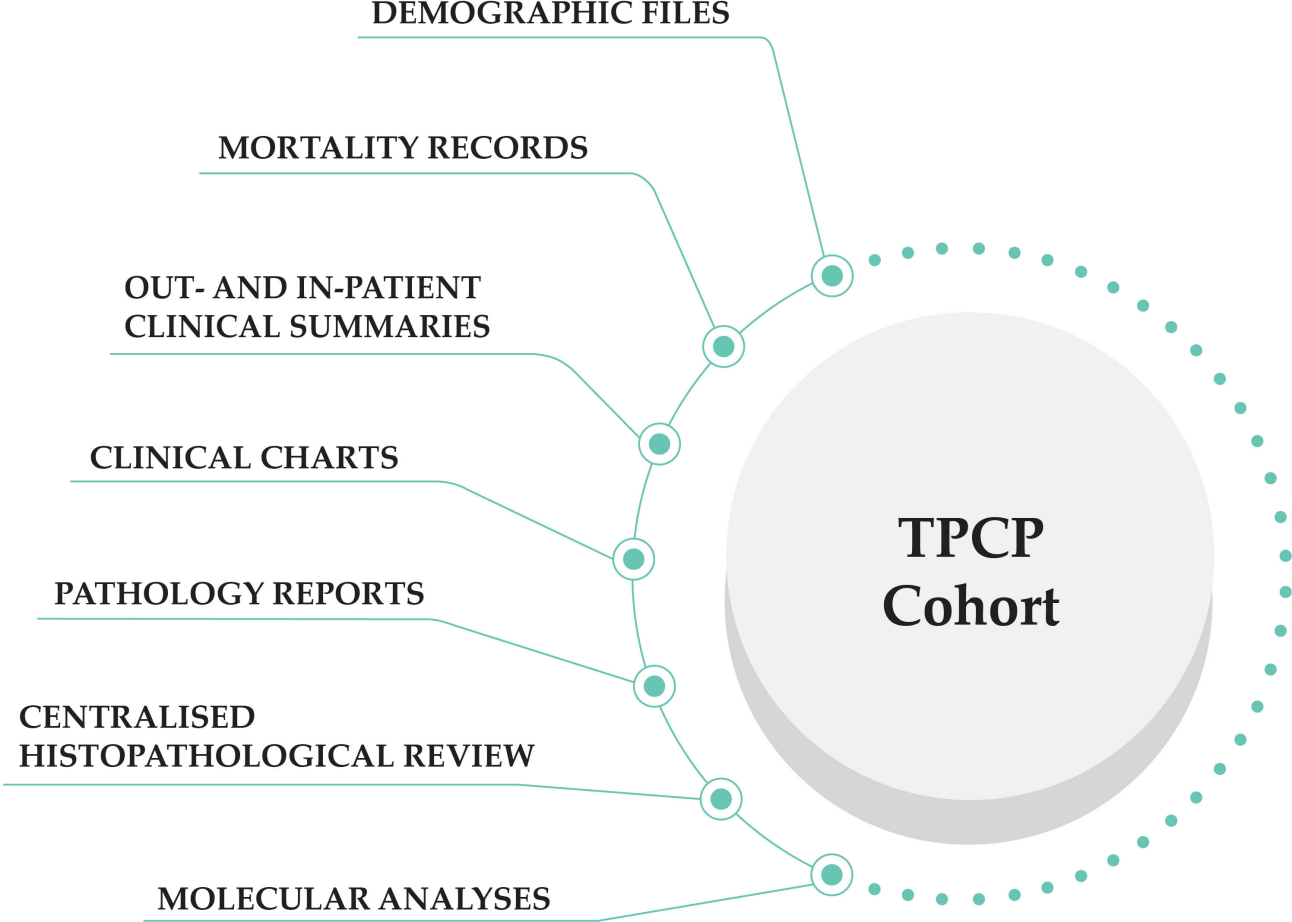
Overview of the TPCP cohort study design data sources.

The cohort integrates data from multiple sources, which are summarized in **Figure 1**. A more detailed presentation of the cohort can be found in **Table 1**. We extracted information from pathology reports, clinical charts, out- and in-patient discharge records, and we digitized and reviewed the slides positive for PCa and those negative for PCa but positive for *high-grade prostatic intraepithelial neoplasia* (HGPIN).

**Table 1:** Variable information.

Variable	Source
Patient Information	
Patient municipality	(4)
Age at diagnosis	(4)
CRCI Diseases	(2)
CRCI	(2)
SDI	(4)
Residence-Hospital distance	(4)
Pathology Information	
Biopsy date	(1)
Pathology Division	(1)
Extra-prostatic invasion	(1)
Patient’s Gleason score	(1)
Clinical T	(8)
Core extraction zone	(1)
Presence of tumorat core level	(1)
Core Gleason score	(1)
Number of sampled cores	(1)
Diagnostic Procedures	
Number of previous negative biopsies	(1), (2), (3)
First negative biopsy date	(1), (2), (3)
DRE	(1), (2), (3)
PSA	(1), (2), (3)
TRUS	(1), (2), (3)
CT/PET-CT	(2)
MRI	(2)
Bone Scintigraphy	(2)
Post-diagnosis Treatment	
Biopsy	(1)
Biopsy date	(1)
TURP	(1)
TURP date	(1)
Prostatectomy	(1)
Prostatectomy date	(1)
Post-prostatectomy Gleason score	(1)
Pathological TNM	(1)
Radiotherapy	(2), (3)
Hormonotherapy	(2), (3)
Follow-up Information	
Vital status	(4)
Cause of death	(5)
Date of death	(4)
End of follow-up date	(4)
Metastasis at follow-up	(2), (3), (8)
Metastasis date	(2), (3), (8)
Metastatic Prostate Cancer	(2), (3), (8)
Death Due to Prostate Cancer	(2), (3), (8)
Centralised Pathology Review: Slide Level	
Total core number	(6)
Slide quality	(6)
Positive core number	(6)
HGPIN	(6)
Chronic inflammation	(6)
Acute inflammation	(6)
Centralised Pathology Review: Core Level	
Length	(6)
Area	(6)
Tumour Length	(6)
Tumour Area	(6)
Primary Gleason	(6)
Secondary Gleason	(6)
Gleason 4 percentage	(6)
ISUP Group	(6)
Centralised Pathology Review: Focus Region Level	
Length	(6)
Area	(6)
Presence of tumor	(6)
Atrophy^a^	(6)
Inflammation^a^	(6)
Perineurial invasion^a^	(6)
Extra-prostatic extension^a^	(6)
Intraductal carcinoma^a^	(6)
Ductal carcinoma^a^	(6)
Poorly formed glands^a^	(6)
Cribriform pattern^a^	(6)
Stroma rich^a^	(6)
Atypical intraductal proliferation^a^	(6)
Mucinous^a^	(6)
Acinar^a^	(6)
Signet ring cell^a^	(6)
Sarcomatoid^a^	(6)
Pleomorphic giant cell^a^	(6)
PIN-like carcinoma giant cell^a^	(6)
Small cell^a^	(6)
Neuro-endocrine differentiation^a^	(6)
Gleason elements^a^	(6)
Molecular Data	
Availability of extracted tumorDNA	(7)
DNA methylation level of selected candidate genes	(7)

CRCI, Charlson-Romano Comorbidity Index; SDI, Social Deprivation Index; DRE, Digital Rectal Examination; PSA, Prostate-Specific Antigen; TRUS, Transrectal Ultrasonography; CT, Computed Tomography; PET-CT, Positron Emission Tomography-Computed Tomography; MRI, Magnetic Resonance Imaging; TURP, Transurethral Resection of the Prostate; HGPIN, High- grade Prostatic Intraepithelial Neoplasia; ISUP, International Society of Urological Pathology; PIN, Prostatic Intraepithelial Neoplasia.

a These variables are Boolean and indicate the presence or absence of something.

(1): pathology reports (2), out- and in-patient clinical summaries (3), clinical charts (4), demographic files (5), mortality records (6), centralized histopathological review (7), molecular analyses (8), derived variables combining the multiple sources.

### 2.1 Patients baseline characteristics and follow-up information

**Figure 2:**
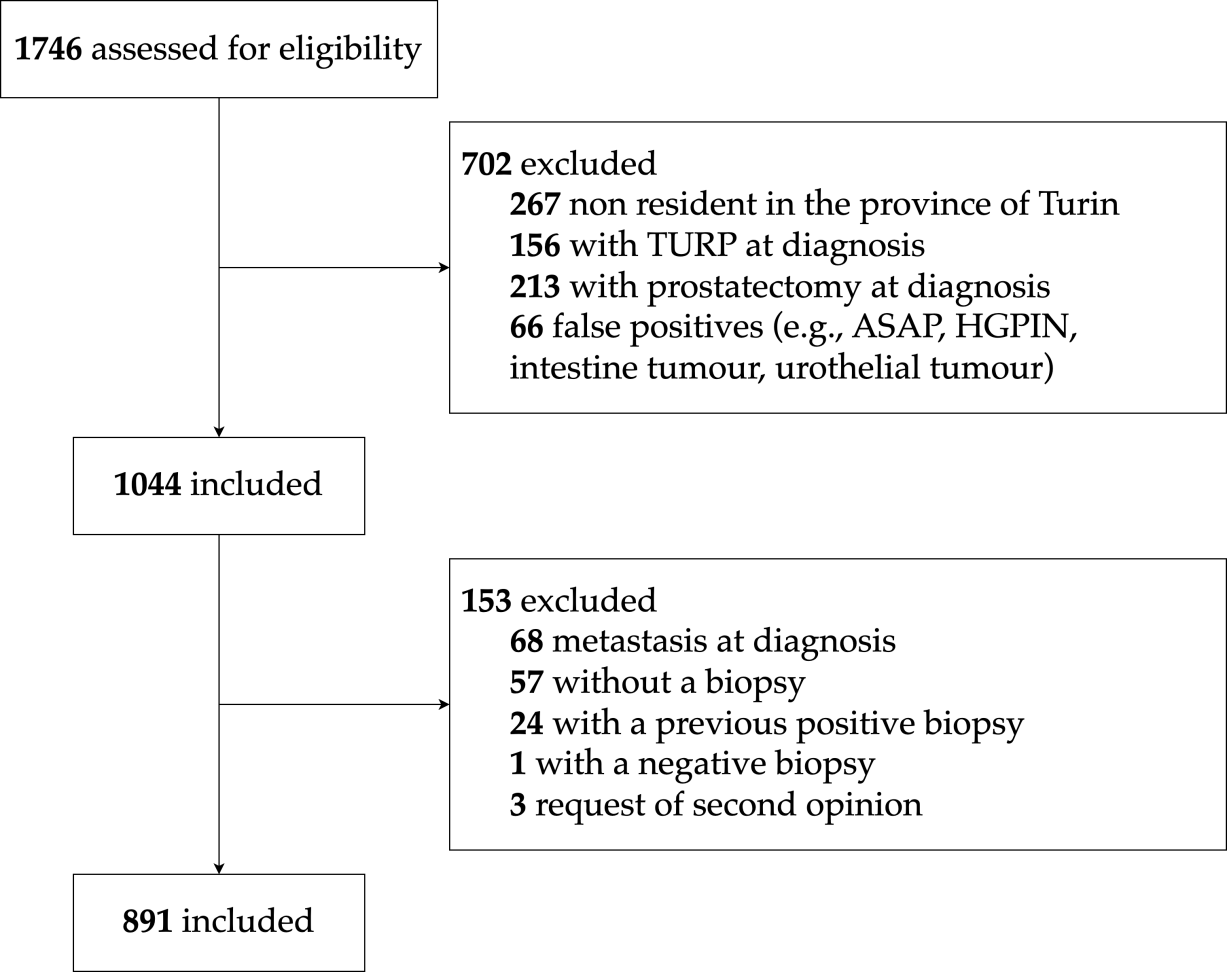
TPCP flow diagram for patient inclusion. ASAP, Atypical Small Acinar Proliferation; HGPIN, High-Grade Prostatic Intraepithelial Neoplasia.

The cohort includes consecutive patients diagnosed with PCa from 1^st^ January 2008 to 31^st^ December 2013, with a prostate biopsy evaluated at one of the two Pathology Divisions of the University Hospital. To be eligible, patients had to be under 85 years of age and without systemic metastases (MX or M0) at diagnosis – based on the available clinical data from the hospital medical charts, pathology reports, and imaging reports. Furthermore, to facilitate the follow-up and to enhance its completeness, we restricted the cohort to residents of the Province of Turin. Moreover, since the TPCP cohort is a biopsy cohort, we excluded patients diagnosed with PCa after *transurethral resection of the prostate* (TURP) or prostatectomy. The patient selection process is illustrated in **Figure 2**: out of 1746 potentially eligible patients diagnosed with PCa during the study period, 891 were included in the final cohort.

Each patient was followed-up from the date of the diagnostic biopsy report until the date of death, emigration outside the Province of Turin, or 31^st^ December 2021, whichever came first. Life-status was assessed through demographic files of the various municipalities, while the specific cause of death was obtained from mortality records held by local health authorities and categorized as PCa-specific mortality or mortality from other causes. The presence of metastasis at follow-up, defined as systematic metastases or involvement of non-regional lymph node(s), was evaluated using hospital information of bone scintigraphy, *computed tomography* (CT) or *positron emission tomography-computed tomography* (PET-CT) exams, discharge and outpatient letters, biopsy and prostatectomy reports, and treatment with abiraterone and/or enzalutamide.

The primary outcome of the study is the occurrence of metastatic PCa, defined as the first recording of metastatic disease after diagnosis; the event date was established as the date of detection of the metastasis. The secondary outcomes of the study are mortality from PCa and overall survival. Among the patients who died from PCa, 7 were reported as metastatic but without the event date and 6 cases had no evidence of metastases in their hospital records. Assuming that lethal PCa always goes through a metastatic stage, the presence and date of metastasis was imputed as follows: for those patients without missing data, we calculated the median *lag-time* between the date of death due to PCa and the date of detection of metastasis (622 days); we then subtracted this lag-time from the date of death of those patients who died due to PCa with no evidence of metastasis (one patient in the cohort died from PCa earlier than 622 days after diagnosis: we used his date of death as the date of metastasis). The same procedure of imputation was used for the 7 cases without the date of metastasis diagnosis. To increase the sensitivity of this procedure, the follow-up for death from PCa was extended by 6 months after 31^st^ December 2021. This allowed the identification of men who had a metastatic disease before the administrative end of the follow-up but died from PCa thereafter (1 over 891).

The cohort data is currently collected and managed in pseudonymized databases using the *Research Electronic Data Capture* (REDCap) platform (21, 22), where random unique personal identifiers (IDs) still allow linking to the personally identifying information.

The study was approved by the University Hospital Ethical Committee (N. 595/2020).

### 2.2 Variable collection

For each patient we extracted the following clinical and pathological information (**Table 1**): age and address at diagnosis, clinical stage, level of pre-sampling PSA, assigned primary and secondary Gleason grade, corresponding Pathology Division, and detailed information on the cores (e.g., number of sampled cores, extraction zone of each core, etc.). To ensure uniformity in the analysis with data from the centralized histopathological review, we converted the Gleason score to the *International Society of Urological Pathology* (ISUP) grade group. Previous negative biopsies were reported for the 11 years prior to the diagnosis. We obtained information on comorbidities from the discharge diagnoses available at the University Hospital from up to 5 years prior to the date of the diagnostic biopsy report – assuming that, as the University Hospital includes most medical specialties, they approximate well the complete 5-year patient history of hospital admissions. Those diagnoses were used to calculate the *Charlson-Romano Comorbidity Index* (CRCI) (23), which is a weighted scoring system that estimates the burden of the following 17 groups of diseases for each patient: any malignancy (including lymphoma and leukaemia), chronic pulmonary disease, cerebrovascular disease, diabetes with and without complications, mild to moderate diabetes, metastatic solid tumor, myocardial infarction, congestive heart failure, renal disease, peripheral vascular disease, rheumatologic disease, mild liver disease, moderate or severe liver disease, peptic ulcer disease, hemiplegia or paraplegia, dementia, and AIDS. Patients with no previous hospital admissions in the 5 years before the prostate biopsy were considered without comorbidities but were treated as a separate category for the CRCI.

Information on the total number of cores was obtained from the pathology reports. For those instances where the information was incomplete, the total number of cores was quantified by reviewing the slides. If the slide was not available, we visually inspected the corresponding *formalin-fixed paraffin-embedded* (FFPE) tissue block and assigned the cores’ number after visual inspection. First, we assigned the total number of cores for 157 slides with fragmented/shattered cores through an educated guess based on the number of cores of the other slides of the same patient as a reference. Second, for 17 slides for which that guess was impossible, we imputed the number of cores based on both the year and the hospital Pathology Division of diagnosis.

Through information contained in the demographic files and publicly available census data, we assigned each patient a *Social Deprivation Index* (SDI) value, available for the whole country at the census level (24). Deprivation indices can represent a proxy for individual deprivation and/or contextual deprivation, and in Italy they have been constructed using census variables.

For each patient, we obtained detailed information on the diagnostic procedures, including whether they had undergone *digital rectal examination* (DRE), *transrectal ultrasonography* (TRUS), CT, PET-CT, *magnetic resonance imaging* (MRI), and bone scintigraphy.

We also collected post-diagnosis information on treatment types and dates, including: TURP, post-diagnosis biopsies, radiotherapy, hormonotherapy, prostatectomy, and the assigned Gleason grade in the corresponding pathology report. For radiotherapy and hormonotherapy, we considered the first visit and the first prescription dates, respectively.

### 2.3 Clinical tumor stage

The tumor extension (cT) is a key marker used in most PCa prognostic models (for convenience, the current version of the cT staging system is summarized in **Supplementary Table S1**). This was reported in only 11% of the pathology reports and was rarely available in the discharge and out-patient letters. Therefore, we derived the three main cT categories based on the combination of DRE, MRI and TRUS, and on whether there was an extracapsular extension. Specifically, whenever information on DRE was available, this information was used to classify the tumor as being clinically apparent or not. For patients for whom information on DRE was not available (147 over 891), we used imaging (either MRI or TRUS) to determine whether the tumor was clinically apparent or inapparent. For patients who did not provide any information on DRE, MRI, or TRUS, but had information on the clinical charts regarding the clinical stage (5 out of 891), we imputed the DRE information as follows: at least cT2, positive DRE; less than cT2, negative DRE. In detail, clinical stage was classified as follows: (i) cT1, a clinically inapparent tumor; (ii) cT2, a clinically apparent tumor confined within the prostate; (iii) cT3, a tumor that extends through the prostatic capsule.

To classify the tumors into the cT subcategories we added information on PSA and used the descriptions available in the pathology reports (instead of clinical DRE findings, which were rarely available for the substages) to understand whether the tumor involved both lobes or – if not – whether it involved more or less than half of one lobe. Specifically, we defined the substages as follows: (i) cT1c, an incidental histological finding; (ii) cT2a, less than 50% of the different prostate regions of a given lobe (but not both) from which cores were extracted and evaluated had at least one positive core; (iii) cT2b, more than 50% of the different prostate regions of a given lobe (but not both) from which cores were extracted and evaluated had at least one positive core; (iv) cT2ab, an undetermined percentage of different prostate regions of a given lobe (but not both) from which cores were extracted and evaluated had at least one positive core, or the cores were extracted from only one region per lobe and positivity was found only in one lobe; (v) cT2c, at least one prostate region of both lobes from which cores were extracted and evaluated had at least one positive core; (vi) cT3+, extracapsular extension.

### 2.4 Digital Pathology Platform and centralized histopathological review

**Figure 3:**
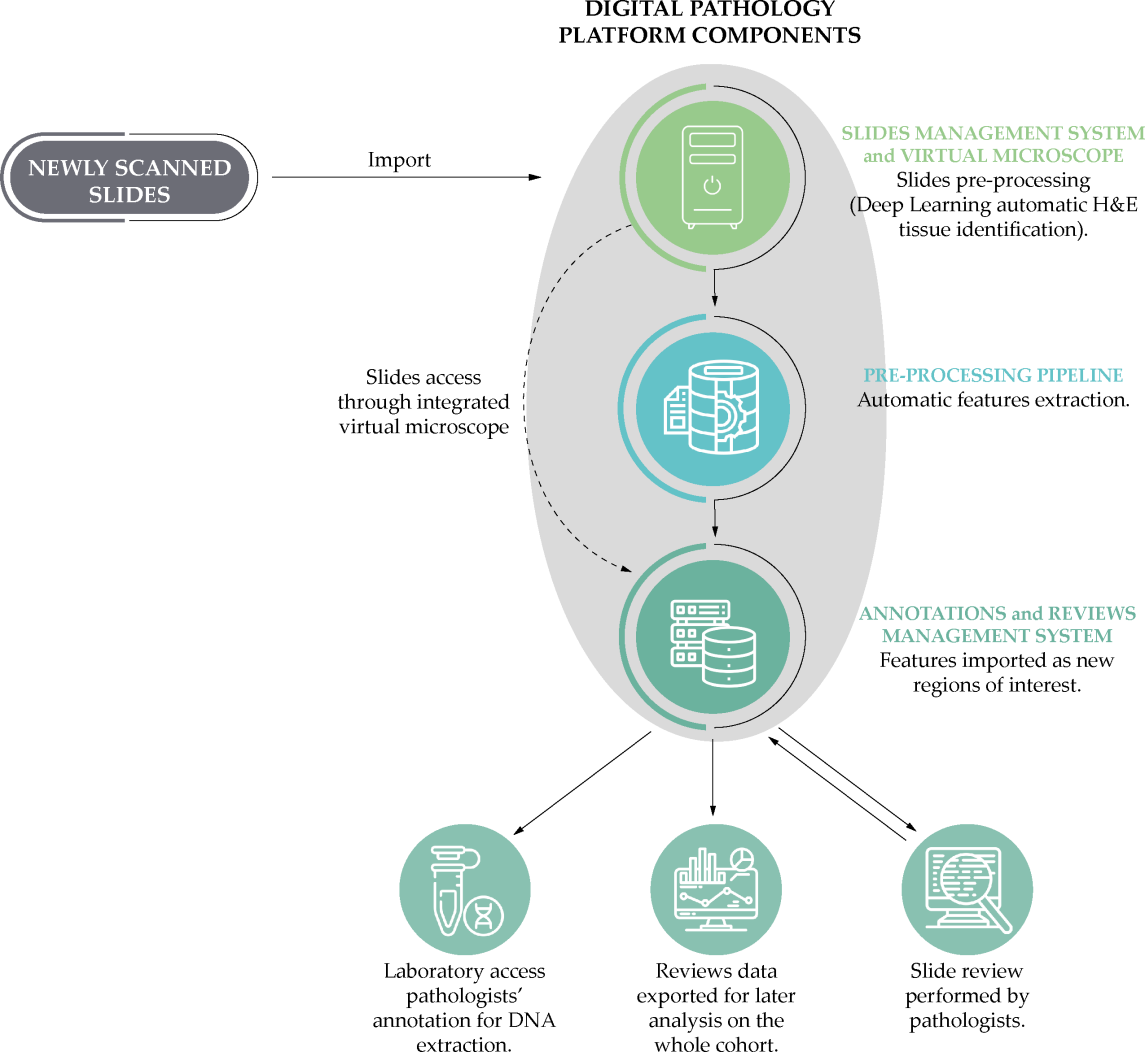
A simplified schematic representation of the analytical process based on the Digital Pathology Platform: from the scanning of the slides to the phases of annotation by the uropathologists and the laboratory post-review.

We digitized all tissue slides that were both positive for PCa and those negative for PCa, but positive for HGPIN according to the original pathology reports, using a NanoZoomer S210 Digital slide scanner (Hamamatsu Photonics K.K., Shizuoka, Japan) at 40x magnification and a scanning resolution of 0.23 µm/pixel. The slides were then reviewed by two uropathologists using the *Digital Pathology Platform* (DPP) (25), created by the *Centre for Advanced Studies, Research and Development in Sardinia* (CRS4) for the tasks of managing, examining and annotating high volumes of high-resolution *whole slide images* (WSI) within the context of clinical research. The system, which has already been used to support other work (26) and previous studies (27), has been demonstrated to be interchangeable with light microscopy (28), and provides automated slide analysis features to improve the time and quality of image annotations (29). An overview of the analytical process using the DPP can be found in **Figure 3**.

The uropathologists have performed a three-level review, evaluating: (i) the slides; (ii) the cores included in each slide; and (iii) specific tissue areas in each core. Concerning the latter, they evaluated all tumor areas and identified, for each slide, two tissue area *focus regions* (FRs): (i) the most representative tumor FR (i.e., the largest of all the regions with the highest Gleason grade), which is also the target for tumor DNA extraction and computational analyses; and (ii) one representative non-neoplastic FR (i.e., the largest area with a distance of at least 1.5 mm from the tumor cells, excluding areas of prostatic intraepithelial neoplasia).

A summary of the histopathological features of interest is reported in **Table 1**. For each slide, the uropathologists reported the quality (high, low but still eligible for review, or ineligible for a meaningful review), as well as the number of positive cores, presence of HGPIN, and acute or chronic inflammation. The DPP automatically inserted annotations identifying the tissue areas on the slide, which the uropathologist then confirmed or corrected. At the core level, the uropathologists reported several key characteristics, including core length, length of the tumor, primary and secondary Gleason scores, percentage of Gleason 4, and the ISUP grade group. In cases (92 over 891) where the image quality was insufficient according to the centralized histopathological review (e.g., vanishing H&E staining), the ISUP grade reported in the clinical records was utilized instead (see **Table 2**). The core area was automatically calculated by the DPP based on the microns-per-pixel ratio of the digitized slide. For each FR the following variables were recorded: length and area, presence of atrophy, inflammation, perineural invasion, extra-prostatic extension, intraductal or ductal carcinoma, presence of poorly formed glands, cribriform pattern, stroma rich, atypical intraductal proliferation, mucinous, acinar, signet ring cell, sarcomatoid, pleomorphic giant cell, PIN-like carcinoma, small cell, neuroendocrine differentiation. In addition, using the tools provided by the DPP, the selected FRs were automatically measured to assess the total area of the tumor. We measured an average area of positive FRs of 6.7 mm^2^ per patient; on the other hand, the average number of positive FRs per patient is 4.6.

**Table 2:** Baseline characteristics of interest for the TPCP cohort.

	TPCP Cohort	
	*n =* 891	%
Pathology Division		
Division I	556	62.4
Division II	335	37.6
Age at Diagnosis (years)		
Median (IQR)	70 (64, 74)	–
Year of Diagnosis		
2008 – 2010	539	60.5
2011 – 2013	352	39.5
PSA (ng/mL)		
Median (IQR)	6.7 (5.0, 10.0)	–
*Missing*	23	–
Previous Negative Biopsies		
0	702	79.7
1	130	14.8
≥ 2	49	5.6
*Missing*	10	–
Number of cores samples at biopsy		
Median (IQR)	14.0 (12.0, 17.0)	
ISUP Grade (based on the pathology reports)		
1	276	31.7
2	293	33.6
3	173	19.9
4	71	8.2
5	58	6.7
*Not evaluable^a^*	20	–
ISUP Grade (based on the centralized histopathological review)		
1	125	14.2
2	289	32.7
3	167	18.9
4	222	25.1
5	80	9.1
*Not evaluable^a^*	8	–
Comorbidities^b^		
Myocardial Infarction	21	2.4
Congestive Heart Failure	13	1.5
Peripheral Vascular Disease	21	2.4
Cerebrovascular Disease	15	1.7
Chronic Pulmonary Disease	24	2.7
Liver Disease (Mild)	9	1.0
Diabetes (Mild to Moderate)	19	2.1
Renal Disease	16	1.8
Any Malignancy (including Lymphoma and Leukaemia)	44	4.9
Metastatic Solid Tumour	9	1.0
Other^c^	5	0.6
Charlson-Romano Comorbidity Index		
0	447	50.2
1	36	4.0
2	51	5.7
≥3	35	3.9
*Without previous admission*	322	36.1
Digital Rectal Examination		
Negative	392	52.7
Positive	352	47.3
*Missing*	147	–
Imaging		
Positive TRUS^d^	184	20.7
Positive MRI^e^	50	5.6
Primary Radical Treatment (within six months)		
Radical Prostatectomy	361	40.5
Radical Prostatectomy & Radiotherapy	36	4.0
Radical Radiotherapy	137	15.4
Deferred^f^	373	41.9
Other Treatments (within six months)		
Androgen Deprivation Therapy	124	13.9
Main cT		
cT1	366	44.5
cT2	288	35.0
cT3	169	20.5
*Missing*	68	–
Detailed cT		
cT1c	366	44.5
cT2a	106	12.9
cT2b	39	4.7
cT2ab	39	4.7
cT2c	104	12.6
cT3+	169	20.5
*Missing*	68	–
Social Deprivation Index		
Low	280	31.4
Medium	280	31.4
High	331	37.1

IQR, Interquartile Range (25th, 75th percentile); PSA, Prostate-Specific Antigen; ISUP, International Society of Urological Pathology; MRI, Magnetic Resonance Imaging; TRUS, Transrectal Ultrasonography.

a ISUP score for patients with a tumor area of less than 5% was not evaluable.

b Each patient in the study population may have experienced multiple comorbidities or no comorbidities (n = 447).

c Comorbidities with a prevalence of less than 1%: Dementia, Rheumatic Disease, Peptic Ulcer Disease, Diabetes (with Chronic Complications), Hemiplegia or Paraplegia, Liver Disease (Moderate to Severe), HIV/AIDS.

d Positivity as reported in the clinical records. All remaining patients had a reported negative TRUS, or no information reported in their records regarding the TRUS.

e Positivity as reported in the clinical records (i.e., suspected abnormal area). All remaining patients had a reported negative MRI, or no information reported in their records regarding the MRI.

f Deferred treatment includes active surveillance or watchful waiting.

Percentages may not total 100% because of rounding.

### 2.5 Cohort analyses protocol

Here we present the protocols for conducting computational histopathology, molecular analyses, and statistical analyses, which will serve as essential frameworks for our future investigations. These protocols outline the systematic procedures and methodologies that will be employed to extract and analyze critical features from digitized slides, study DNA methylation patterns, and develop prognostic models to assess the progression and outcomes of PCa.

#### 2.5.1 Computational histopathology

The protocol for the extraction of features from the digitized slides makes use of Deep Learning models. Specifically, the selection of suitable slides for agnostic feature extraction is based on overall image quality reported by the uropathologists: in total, 84.9% of slides are reported to be suitable for the analyses (1928 over 2272). Adequate slides are first subject to a color normalization step to correct color fluctuations that usually exist in WSI. Then, for each slide, one or more FRs are selected, based on traits derived from the slide review process (e.g., the presence of tumor), and used as masks for the identification of image areas for the extraction of small image subregions with a fixed pixel resolution at a fixed magnification level (*patches*). After the extraction, the patches are filtered to exclude those unsuitable for the analysis process (e.g., low tissue content). Each patch has associated metadata, which are produced during their extraction from the WSIs, like tissue coverage ratio, tissue status (e.g., tumor or non-tumor), Gleason score (only available for those belonging to a positive FR), patch resolution, and magnification level used for extraction. The per-patient average number of patches extracted from positive FRs is 668.

To extract feature vectors from the patches, the study protocol envisages the use of *Variational Autoencoders* (VAs), a class of Deep Neural Networks consisting of two main blocks of networks: an *encoder* and a *decoder* (30). These are designed to: (i) perform the encoding of the input data into a lower dimensional embedding, and (ii) reconstruct the input from the lower dimensional space. The main goal of VAs is to obtain a latent representation of the data, and extract features from the images. The extraction of patches and the generation of feature vectors is executed on every available FRs for each patient, including the one identified for DNA extraction. The autoencoder representation features will be included as covariates in the final overarching model. Furthermore, the extracted patches will remain accessible for exploration with other methods.

#### 2.5.2 Molecular analyses

We selected seven candidate genes for the analysis of DNA methylation: *GSTP1* (Glutathione S-Transferase P-1), *APC* (Adenomatous Polyposis Coli), *LINE-1* (Long Interspersed Nuclear Element-1), *PITX2* (Paired-like homeodomain transcription factor 2), *ABHD9* (Abhydrolase domain containing 9), *Chr3-EST* (Expressed sequence tag on chromosome 3) and *GPR7* (G protein-coupled receptor 7). *LINE-1* was selected as a proxy for global DNA methylation status. The remaining six genes, on the other hand, were selected through an extensive review of the literature to identify genes for which methylation in the tumor tissue was found to predict PCa progression in at least two studies including at least 200 patients, and, possibly, an external validation (31–33). The search-string that was used for the review process in PubMed (last updated March 1^st^ 2023) is reported in the **Supplementary Table S2** (15, 17, 30–43).

The DNA extraction protocol foresees the extraction from the patient’s FR with the highest Gleason score and the largest tissue area. FRs shorter than 1 mm are excluded to avoid contamination from the adjacent non-tumor tissue. Three to five sequential sections (10 µm thick) are cut from the corresponding FFPE tissue block. The region is scraped with a sterile scalpel and both the subsequent extraction and purification are carried out using QIAamp DNA FFPE Tissue (Qiagen GmbH, Hilden, Germany), which was found to be superior to other extraction kits in a recent study on FFPE prostate biopsies (44). The DNA extraction rate was found to be ≥99% on the first 426 samples (to date 07/08/2023).

The protocol for methylation analyses involves a bisulfite modification using the EpiTect bisulfite kit (Qiagen, Hilden, Germany). Then, the modified genomic DNA is used immediately for methylation analysis or stored at −80°C. The methylation level of selected genes is measured using QX200 Droplet Digital PCR System (Bio-Rad, California, USA), ensuring high sensitivity and specificity without the use of standard curves for absolute quantification. Fluorescence data is analyzed using the QuantaSoft™ Analysis Pro Software and the results are reported as methylation percentages. Each run includes positive controls with known methylation percentage and no-template control. Primers and probes sequences and PCR conditions for each gene are reported in **Supplementary Table S3**).

#### 2.5.3 Statistical analyses

The study’s primary outcome is the occurrence of metastatic PCa. The main secondary outcomes of interest are mortality from PCa and overall mortality. We are also interested in identifying predictors of treatment strategies and considering the role of treatment in the prognostic models (45). Finally, the baseline characteristics of the patients at diagnosis can be analyzed cross-sectionally to identify associations among the clinical, non-clinical, molecular, and histopathological predictors. For example, the integrated data of the TPCP cohort could allow the exploration of the link of the histological characteristics assessed by the clinicians with both the histopathological features extracted from the digitized slides and the DNA methylation tumor profiles.

The analysis plan for prognostic modelling involves sequential steps. First, the best existing prognostic models [including MSKCC, CAPRA, PREDICT (46), Survival Quilts (47)] are adapted to the TPCP cohort data. Second, the updated models are extended by adding, separately, additional patient characteristics (e.g., comorbidities, socioeconomic position), the histological characteristics assessed by the clinicians, the molecular markers, the histopathological features extracted from the digitized slides; the performances of these extended models are assessed in terms of calibration and discrimination. Third, all relevant predictors, irrespective of their source, are included in a final overarching model. For both the metastatic PCa and the PCa mortality outcomes, models consider mortality from other causes as a competing risk. The different models are described and compared in terms of calibration and discrimination.

All prognostic models are validated internally and, whenever possible, externally. As the TPCP cohort includes cases from two different Pathology Divisions, it should be possible to compare them to further validate the models.

## 3 Descriptive results

The TPCP cohort includes 891 PCa patients, with a median follow-up duration of 10 years, and a maximum duration of 14 years: 97 patients developed metastatic disease during the follow-up and 301 patients died; of these, 56 died from PCa (**Table 3**). The baseline descriptive data of the cohort are provided in **Table 2**, in terms of absolute numbers, proportions, median and interquartile range (IQR), and distributions. Almost three-quarters of the patients were 65 years of age or older at diagnosis, the median PSA was 6.7 ng/mL, and approximately 45% had a cT1 stage disease. For almost 42% of the patients, there were no in- or out-patient admissions for prostatectomy and radiotherapy within the first six months after diagnosis.

**Table 3:** Primary and secondary outcomes in the TPCP cohort.

	TPCP Cohort
	*n =* 891
Follow-up Time (years)	
Median (IQR)	10.4 (8.2, 12.9)
Primary Outcome	
Metastatic Prostate Cancer	97
Secondary Outcomes	
Death from Prostate Cancer	56
Death from Any Cause	301

IQR, Interquartile Range (25^th^ – 75^th^ percentile).

**Figure 4:**
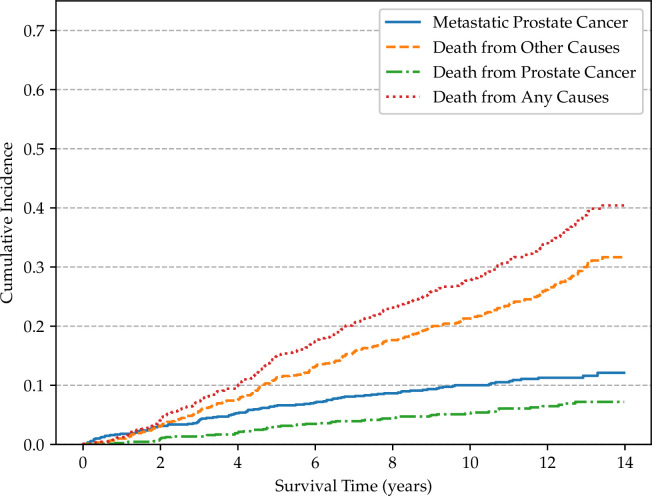
Cumulative incidence functions for the TPCP cohort (*n* = 891).

**Figure 5:**
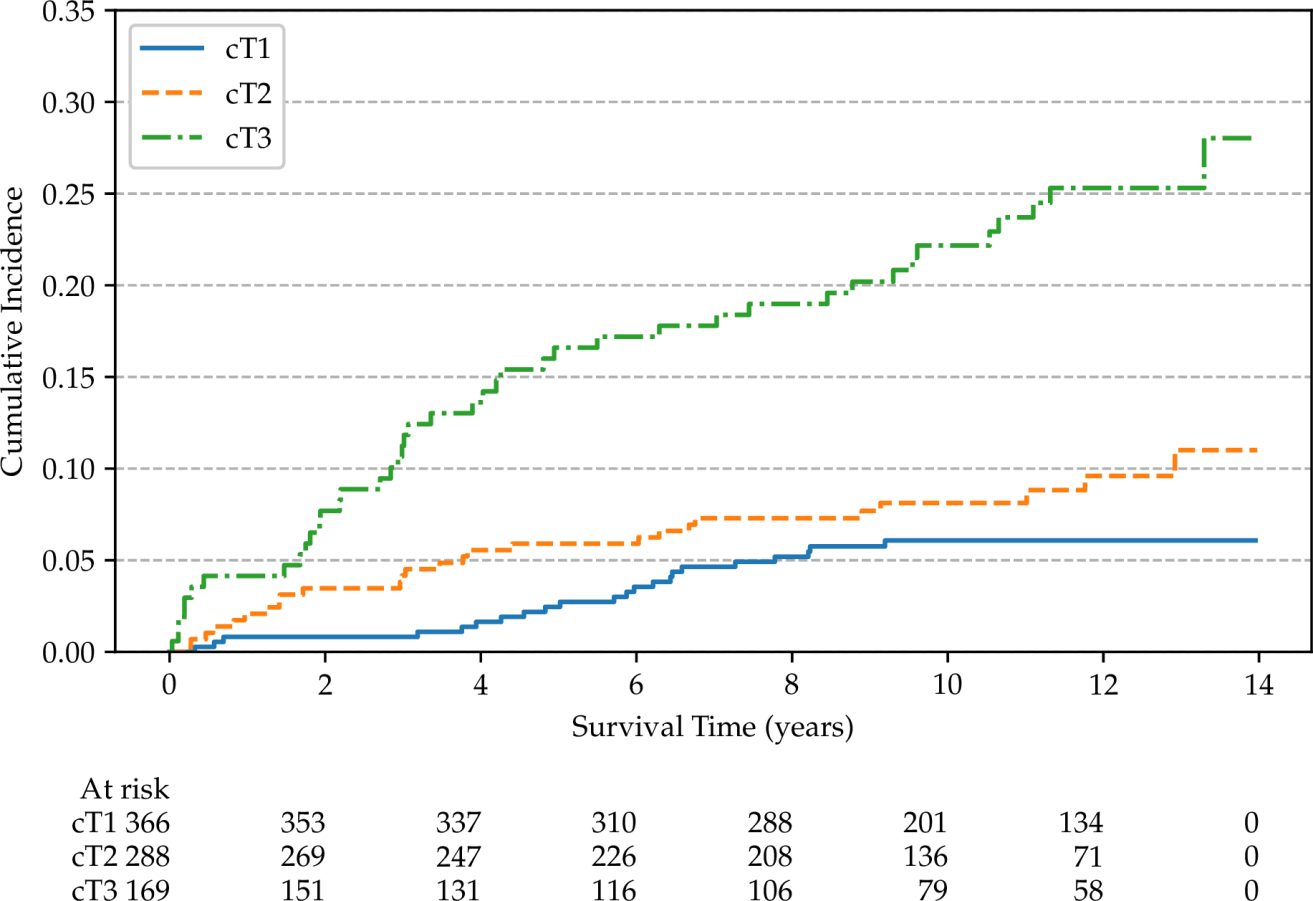
Non-parametric cumulative incidences of metastatic prostate cancer, by clinical stage (*p* < 0.001).

**Figure 6:**
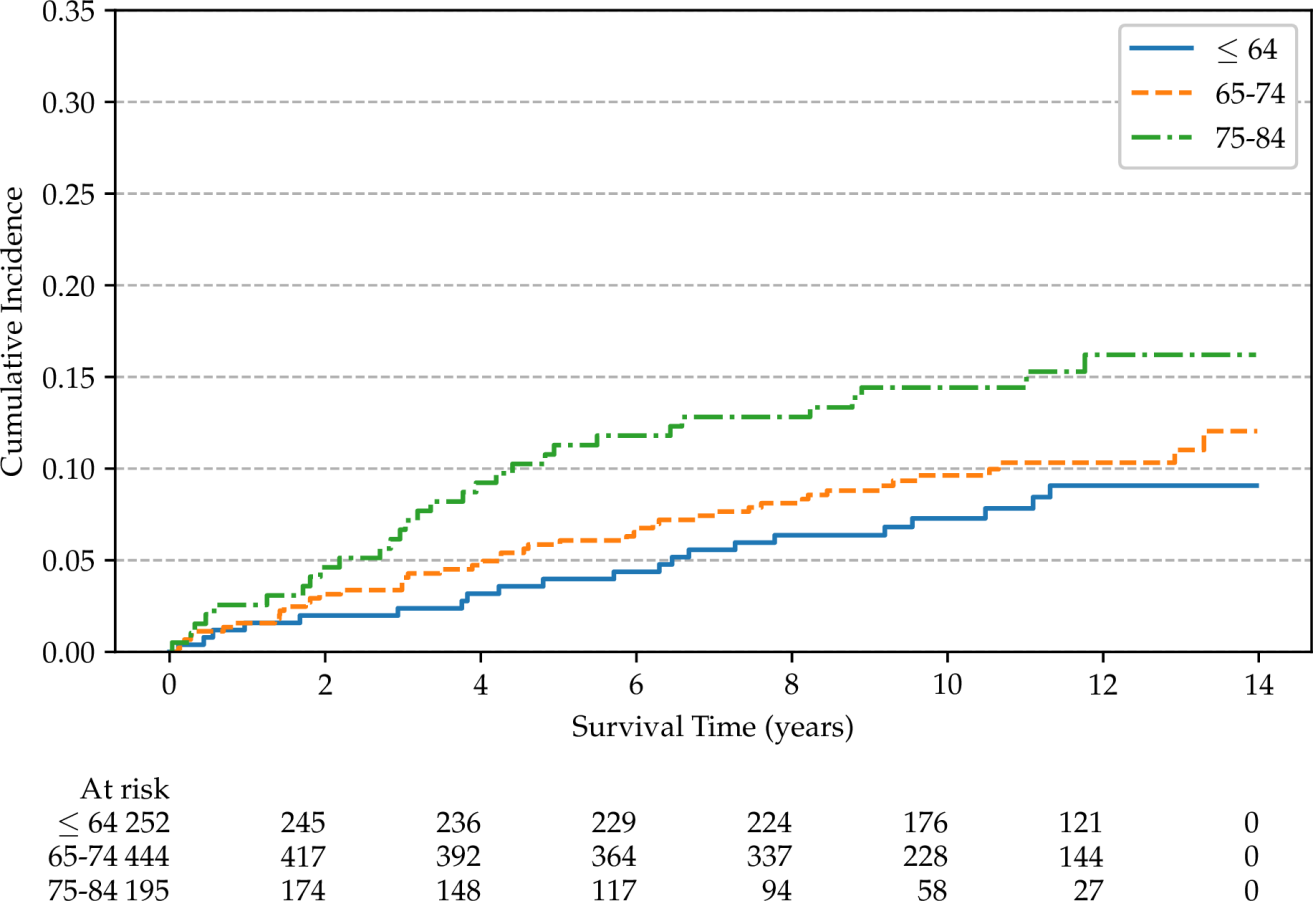
Non-parametric cumulative incidences of metastatic prostate cancer, by age (*p* = 0.045).

**Figure 7:**
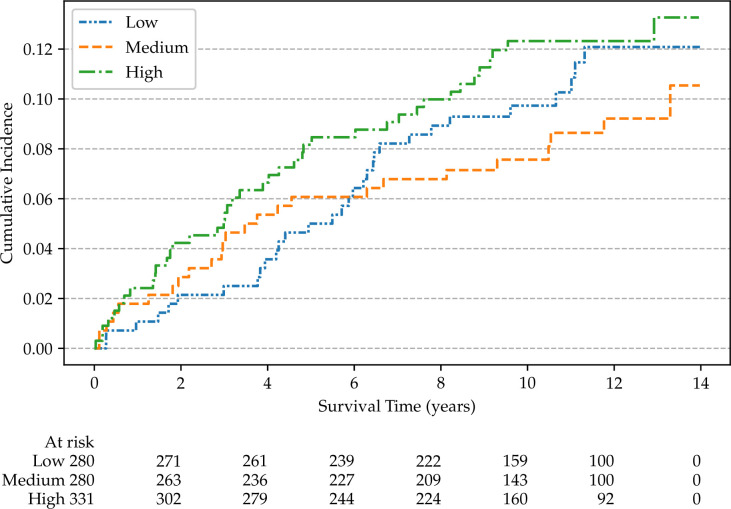
Non-parametric cumulative incidences of metastatic prostate cancer, by social deprivation index (*p* = 0.40).

Non-parametric cumulative incidence curves are calculated for metastatic disease and mortality from PCa, and potential differences according to patients’ characteristics are tested using Gray’s test (48). **Figure 4** reports the overall cumulative 14-year incidences of metastatic PCa (12.1%), lethal PCa (7.2%) death from other causes (31.7%), and overall mortality (40.4%). The cumulative incidences of metastatic patients, stratified by cT stage, age, and SDI are shown in **Figures 5**–**7**, respectively. Using mortality from PCa instead of metastatic disease as the outcome yielded similar results. Men with an advanced cT stage and older age had a poorer prognosis, with a 14-year incidence of metastatic disease of 6.1% for cT1, 11.0% for cT2, and 28.0% for cT3. The 14-year incidence of metastatic disease differed across age groups, with rates of 9.1% for patients less than 64 years of age, 12.0% for those between 64 and 74 years of age, and 16.2% for those older than 75 years of age. There was no clear evidence of association between SDI and cumulative incidence of metastatic disease, although the latter was higher in patients residing in the most socially deprived areas, who had also a much higher overall mortality compared to the other patients (**Supplementary Figure S1**). Among the 68 men excluded from this study due to metastatic disease at diagnosis (M1), the 14-year mortality from PCa was 63.2%.

## 4 Discussion

Thanks to a collaboration of different institutions from a multidisciplinary team, including epidemiologists, biostatisticians, molecular biologists, uropathologists, bioinformaticians, urologists, radiation and medical oncologists, and computer scientists, we have established the TPCP cohort, a relatively large historical biopsy-cohort of consecutive unselected PCa patients, all diagnosed in a single institution with a long-term follow-up for lethal disease. This cohort integrates several sources of information and will support both calibration and validation of existing prognostic models, as well as the development of new ones. The selection of patients, the choice of methodology, the selection of prognostic markers, and the composition of a multidisciplinary research group were decisions taken with the aim of improving the feasibility of the clinical translation of the prognostic models.

With the integrated data from the TPCP cohort we will be able to explore the links between the patients’ characteristics assessed by the clinicians, the histopathological features extracted from the digitized slides and the methylation profiles in the PCa tissue: this work will potentially enable us to link the histopathological features with the epigenetic characteristics to understand the meaning of the former and, consequently, improve their interpretation.

Our approach has some limitations. First, we relied on retrospective data available in a single institution, as we could not access data for patients who were diagnosed at the study University Hospital but were followed-up elsewhere. This limitation implies that information on post-diagnostic variables, including the presence of metastasis, is obtained with high specificity but a lower sensitivity. We expect, however, that the lack of clinical post-diagnostic information has a low impact on the quality of the TPCP cohort data, as: (i) we restricted the cohort to those patients who were resident in the Province of Turin; (ii) the University Hospital is the main institution for treating PCa in the Piedmont Region; and (iii) all patients were initially diagnosed at the University Hospital. The follow-up for overall and PCa-specific mortality (i.e., the study secondary outcomes) is instead complete for all cohort members, as we obtained this information from the demographic files. We used the information on PCa mortality to impute the presence of metastasis for patients who did not have this information recorded in their hospital clinical records. It follows that we have a very good level of completeness also for metastatic disease.

The fact that the TPCP cohort is based on a single institution simplifies the harmonization of clinical and histopathological variables but may also limit the external validation. However, it should be noted that the cohort obtained the biopsies from two Pathology Divisions of the University Hospital – which are linked to two different Urology Divisions. Thus, the respective subsets of the cohort can externally validate each other. Furthermore, we will seek collaboration with other existing cohorts in the future for proper external validation.

To ensure a long duration of follow-up, we only included patients diagnosed before 2015 – before the widespread adoption of *multi-parametric MRI* (mpMRI), which impacts on the number and type of patients who undergo a biopsy for suspect PCa (49). However, it is important to note that the main clinical contribution of the use of mpMRI is the reduction of unnecessary biopsies of benign prostate tissue or indolent tumor, most of which should be classified as low risk by the models we have discussed. Therefore, the prognostic models that will be developed in our study could be adapted to a context of patients pre-selected through mpMRI. However, we are unable to incorporate mpMRI radiomics in our prognostic approach directly. We acknowledge that further research will be required to study the potential contribution of mpMRI radiomics using more recent cohorts.

### 4.1 Using the TPCP cohort for external validation

To explore the possibility of using the TPCP cohort data for the replication and external validation of a prognostic model for PCa, please contact Lorenzo Richiardi (lorenzo.richiardi@unito.it), the Principal Investigator for the TPCP cohort, for further questions about data access. Further information about data access is provided in the Data Availability Statement.

## 5 Conclusion

This work presented the established TPCP biopsy cohort of almost 900 PCa patients followed for a median time of 10 years. We have collected and analyzed clinical and pathological information from numerous clinical and demographic data sources. The initial evaluation of cohort outcomes is consistent with previous studies, with age and clinical stage at diagnosis being important prognostic factors. Further, we have assembled an extensive set of digitized biopsy tissues slides reviewed and annotated by uropathologists. This first set of data is the basis for the ongoing acquisition of molecular and histopathological biomarker data into a single, integrated collection. This collection will feed the statistical analyses described in our protocol to adapt the best current prognostic models for PCa to this cohort, and to study the integration of these molecular and histopathological biomarkers both in the best of these existing models as well as in the development of new prognostic models.

## Data availability statement

The datasets presented in this article are not readily available because of legal and ethical reasons. However, to explore the possibility of using the TPCP cohort data for the replication and external validation of a prognostic model for PCa, please contact Lorenzo Richiardi (lorenzo.richiardi@unito.it), the Principal Investigator for the TPCP cohort, for further questions about data access. Although data access may be difficult in the current legal framework, we are open to supporting data reuse at least by discussing an analysis plan, implementing it in our cohort and sharing the results. We have published a catalogue [accessible on the study’s website (50)] that presents the metadata of the TPCP variables, which should help better understand the data and its potential applicability to new scenarios. It will be regularly updated to ensure the latest information is available. Requests to access the datasets should be directed to Lorenzo Richiardi, lorenzo.richiardi@unito.it.

## Author contributions

LR and DZ were responsible for the study concept and design. ND and LR collaborated on the first draft of the manuscript. LMi, VF, PV, and ND conducted the data collection and organized the database. ND performed the data analysis. MF and FG conducted the histopathological review. LL, MDR, FF, and LP were involved in the development and maintenance of the digital pathology software. VF and PV conducted the molecular analyses. LMo, PC, MP, PG, GC, MO, UR, GI, PF, EI, OA, RZ, and AP critically interpreted the data. All authors contributed to the article and approved the submitted version.
