# Supplementary material for: Cohort profile: the Turin prostate cancer prognostication (TPCP) cohort
Source: Front Oncol. 2023 Oct 6;13:1242639. doi: 10.3389/fonc.2023.1242639 (PMC10587560; doi:10.3389/fonc.2023.1242639)
Supplement: Supplementary file 1 [file DataSheet_1.pdf]

Supplementary Material

1 SUPPLEMENTARY TABLES AND FIGURES

1.1 Tables

Table S1. Clinical Tumour (cTNM) classification of prostate cancer, according to the 8th edition of the Union for International Cancer Control (UICC).

Stage	TPCP Cohort
Primary Tumour	
cTX	Primary tumour cannot be assessed.
cT0	No evidence of primary tumour.
cT1	Clinically inapparent tumour, neither palpable nor visible by imaging.
cT1a	Tumour incidental histological fining in less than 5% of tissue resected.
cT1b	Tumour incidental histological fining in more than 5% of tissue resected.
cT1c	Tumour identified by needle biopsy (e.g., because of elevated prostate-specific antigen).
cT2	Tumour that is palpable and confined within the prostate.
cT2a	Tumour involves one-half of one lobe or less.
cT2b	Tumour involves more than one-half of one lobe, but not both lobes.
cT2c	Tumour involves both lobes.
cT3	Tumour extends through the prostatic capsule.
cT3a	Extracapsular extension (unilateral or bilateral).
cT3b	Tumour invades seminal vesicle(s).
cT4	Tumour is fixed or invades adjacent structures other than seminal vesicle(s).

Table S2. PubMed search string and key words.

**Search date**	01/03/2023
**Number of records**	1459
**PubMed Search String**	((((((prostate[MeSH Terms]) OR prostate) OR prostate neoplasm[MeSH Terms]) OR prostate neoplasm) OR prostate cancer) OR prostate tumor) AND ((((((methylation[MeSH Terms]) OR methylation) OR DNA methylation[MeSH Terms]) OR DNA methylation) OR methylation marker) OR methylation biomarker) AND ((((((((((((((((((prognosis[MeSH Terms]) OR prognosis) OR survival[MeSH Terms]) OR survival) OR mortality[MeSH Terms]) OR mortality) OR death[MeSH Terms]) OR death) OR recurrence[MeSH Terms]) OR recurrence) OR relapse) OR biochemical recurrence) OR progression) OR disease progression[MeSH Terms]) OR disease progression) OR progression free survival[MeSH Terms] OR progression free survival) OR recurrence free survival) OR disease free survival[MeSH Terms]) OR disease free survival) OR PSA free survival)
**Key Words**	prostate neoplasm, prostate cancer, prostate tumor, methylation, DNA methylation, methylation marker, methylation biomarker, prognosis, survival, mortality, death, recurrence, relapse, biochemical recurrence, progression, disease progression, progression free survival, recurrence free survival, disease free survival, PSA free survival

Table S3. Primer and probe sequences, PCR annealing temperatures and primers and probes concentrations. Legend: F, Forward; R, Reverse; M, Methylated; U, Un-methylated.

Gene	Primers 5'-3'	Probes 5'-3'	Annealing Temperature (°C)	Primer Concentrations (nM)	Probe Concentrations (nM)
ABHD9	F: GGTGTTAGGGTTTAGGGGTT<br>R: CCAAATATTTACCTAACACTCAAATA	M: AACTATTTTCTATCGAAACCGCCCG<br>U: AACTATTTTCTATCAAAACCACCCACCTCT	55	450	M: 300<br>U: 250
Chr3-EST	F: TTGTAGGGTTTTTTTTGGGTT<br>R: CTCAAAACCCTTAAAAACATAAA	M: ATAACCACACTACGCGCCTCC<br>U: ATAACCACACTACACACCTCCCACA	55	450	M: 300<br>U: 250
GPR7	F: CATCCCTACACTTCCAAAC<br>R: GGAGTTGTTAGGAGAAAAGTT	M: CGAACACCCAACCGACAAACG<br>U: CAAACACCCAACCAACAAACATCTCA	55	450	M: 250<br>U: 250
GSTP1	F: GATTTGGGAAAGAGGGAAAGGT<br>R: CAAAAAAACGCCCTAAAATCC	M: TAGTTGCGCGGCGATTTCGG<br>U: TAGTTGTGTGGTGATTTTGG	50	450	M: 250<br>U: 250
APC	F: GTAGTTGTGTAATTGGTTGGATG<br>R: CACCAATACAACCACATATC	M: TTCGTCGGGAGTTCGTCGATT<br>U: TTTGTTGGGAGTTTGTTGATT	52	450	M: 250<br>U: 250
PITX2	F: TAGGGGAGGGAAGTAGATGTTA<br>R: CAAATCCCCTCTCCTTTC	M: GAGTCGGGAGTCGGAGTCGGGAGAGCG<br>U: GAGTTGGGAGTTGGAGTTGGGAGAGTG	55	450	M: 250<br>U: 250
LINE-1	F: TTTGAGTTAGGTGTGGGATATAGTT<br>R: CACCTAAAAAATCCAATCACTCC	M: TTCGTGGTGCGTCGTTTTTTAA<br>U: TTTGTGGTGTGTTGTTTTTTAA	55	450	M: 250<br>U: 250

ABHD9: Abhydrolase domain containing 9.
Chr3-EST: Expressed sequence tag on chromosome 3.
GPR7: G protein-coupled receptor 7.
GSTP1: Glutathione S-Transferase P-1.
APC: Adenomatous Polyposis Coli.
PITX2: Paired-like homeodomain transcription factor 2.
LINE-1: Long Interspersed Nuclear Element-1.

1.2 Figures

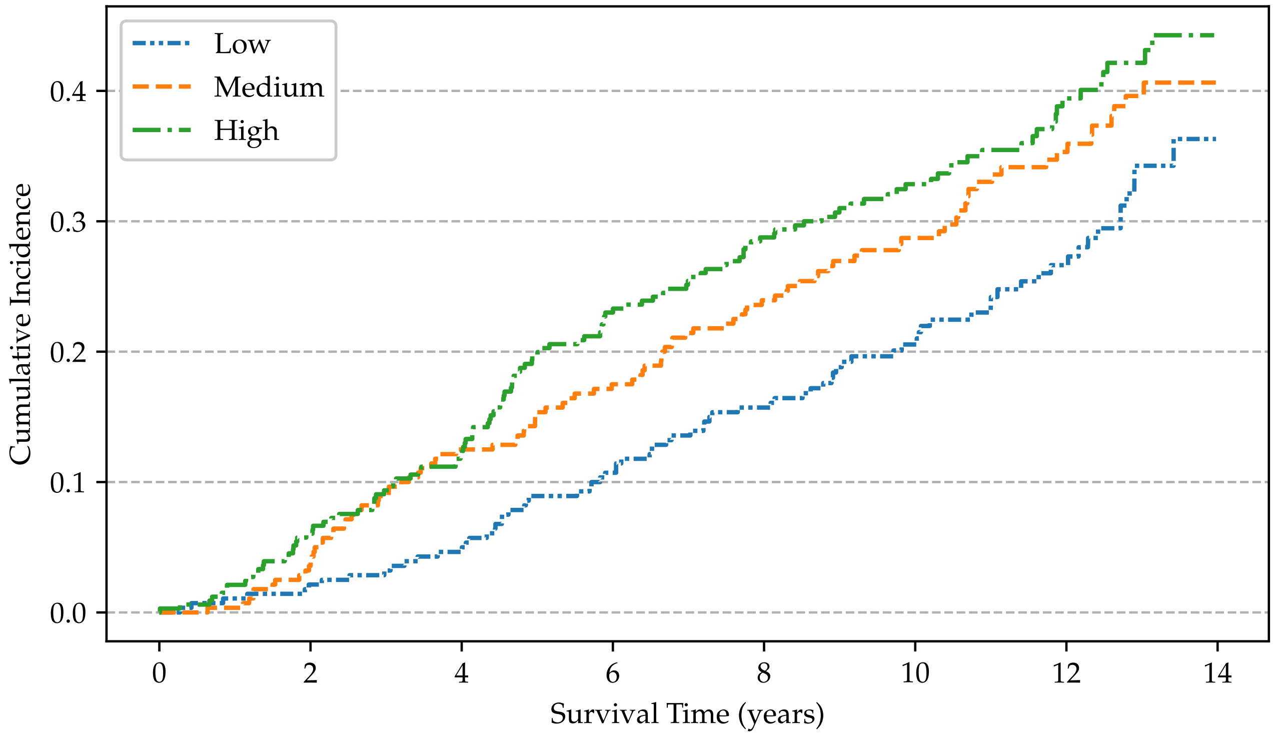

At risk							
Low 280	274	267	250	236	169	110	0
Medium 280	270	245	231	212	147	105	0
High 331	311	290	254	235	171	99	0

Figure S1: Non-parametric cumulative incidences for overall mortality, by social deprivation index (SDI) ($p = 0.008$).
